# Current Applications and Future Directions of Lasers in Endodontics: A Narrative Review

**DOI:** 10.3390/bioengineering10030296

**Published:** 2023-02-26

**Authors:** Qin Huang, Zucen Li, Ping Lyu, Xuedong Zhou, Yi Fan

**Affiliations:** State Key Laboratory of Oral Diseases, National Clinical Research Centre for Oral Diseases, West China Hospital of Stomatology, Sichuan University, Chengdu 610041, China

**Keywords:** minimally invasive dentistry, laser therapy, endodontic treatment, post-operative pain, root canal treatment, dentinal hypersensitivity, pulp capping, pulpotomy

## Abstract

The utilization of lasers has been regarded as a novel technique for the purposes of clinical use in the dental field. Recently, numerous studies have been conducted on the potential applications of laser therapy in endodontics. Moreover, due to their ablation, penetrability, and disinfection capabilities, lasers have performed well with respect to endodontic treatments, including root canal treatment, vital pulp therapy (pulp capping and pulpotomy), dentinal hypersensitivity treatment, and management of dental pain related to pulp and periradicular disease. In particular, the superiorities of laser-aided pulp therapy are emphasized through condensed clinical controlled trials, and histological studies, in this review. Moreover, the ingenious use of laser applications with respect to aiding in the acceleration of root development and the extraction of foreign matters (i.e., broken files and fiber posts) in canals has quickly become the cutting-edge trend of current research. This review offers a summary and discussion of the current literature on all the aforementioned laser applications. Moreover, the characteristics of laser devices, including erbium lasers, neodymium-doped lasers, CO_2_ lasers, and diode lasers, are detailed and discussed here, providing useful references for laser application in endodontics. We also focus on the different wavelengths with respect to the lasers that are applied in endodontics. High-power lasers perform well as operative instruments; in addition, low-level lasers lead to the regulation of pulp inflammation, and the promotion of pulp healing. This narrative review provides a summary of the advanced applications of lasers in conjunction with various devices in the practice of endodontics, and aims to inspire innovative perspectives on lasers in the context of the treatment of dental diseases, especially pulp diseases, in the future.

## 1. Introduction

Endodontic treatment in dentistry involves decimating bacterial contamination, cleansing the root canal system, as well as regaining favorable morphology, and the functions of the tooth [1]. Mechanical and chemical therapies are widely accepted in the clinic for the purposes of decontamination, and preparation during root canal treatment. Ultrasonic devices, handpieces, or rotary instruments are important assistants in the cleaning, shaping, and obturating of root canals [2]. The practice of intracanal medication is performed in the form of root canal dressing for disinfection, and for inflammation reduction [3]. However, the frequent application of drugs, such as calcium hydroxide, mineral trioxide aggregate (MTA), sodium hypochlorite rinsing solution, and other antibacterial agents, may arouse concern regarding antimicrobial resistance [4]. Indeed, mechanical contact between the apparatus and oral tissues could cause unanticipated damage or infection. Moreover, endodontic failure, which is caused by inadequate treatment, always results in persistent intraradicular infection. Furthermore, it was reported that approximately only two-thirds of retreatment cases could reach a successful outcome [5].

Due to their characteristics regarding debridement, ablation, the regulation of inflammation, and the acceleration of healing, the application of lasers with different wavelengths and devices was introduced to dentistry, and has been conducted for decades [6]. Laser therapy has fast become a state-of-the-art practice in respect to maxillofacial surgery, oral implant surgery, periodontics, and endodontics. The first report on its use in periodontics was in the 1980s [7]. Currently, it has been utilized as a frequently used tool in the context of periodontal surgery. A high-power laser could also be applied during the scaling and root planing (SRP) process; additionally, a low-level laser could be used in aiding with the reduction of pathogenic bacteria in periodontal pockets [6]. Furthermore, the application of lasers could also serve as a feasible option for osteotomy, premalignant lesions excision, and periimplantitis treatment [8]. As for endodontics, although numerous studies have been conducted that focused on the advances of laser treatment, a comprehensive review of the role of lasers in various branches of endodontic treatment is still lacking.

**Figure 1:**
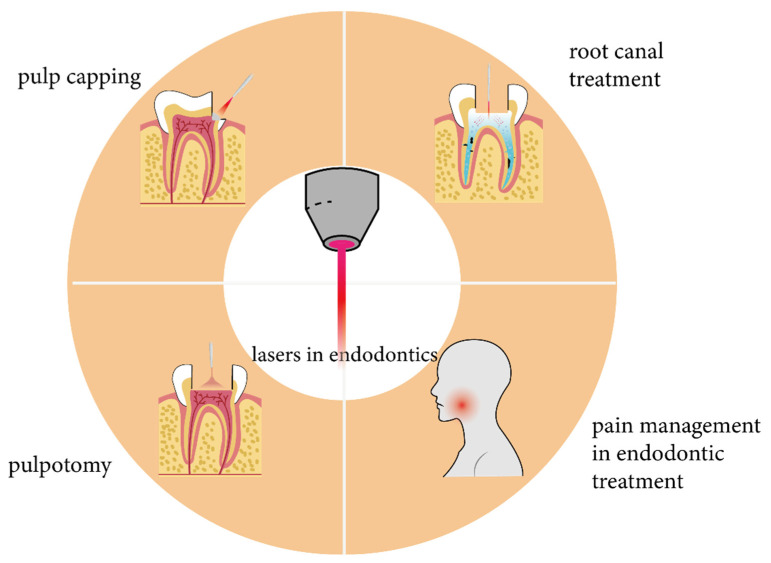
Applications of lasers in endodontics.

In this review, we summarize the advanced applications of lasers in the context of endodontic treatment (Figure 1), including root canal treatment; pulp capping; pulpotomy; dentinal hypersensitivity treatment; and management of dental pain related to pulp and periradicular diseases. Moreover, laser-based technology in respect to root development acceleration, and the removal of broken files and fiber posts, is currently the cutting-edge trend of various research studies. This review is expected to guide the further exploration of novel applications of lasers in the practice of endodontics, thus aiming at satisfying progress demands in clinical practice.

## 2. Types of Laser Systems in Endodontics

The types of lasers commonly used in clinical endodontic treatment include: erbium:yttrium–aluminum–garnet (Er:YAG) lasers; erbium, chromium-doped yttrium scandium gallium garnet (Er,Cr:YSGG) lasers; neodymium-doped yttrium aluminum garnet (Nd:YAG) lasers; neodymium: yttrium–aluminum–perovskite (Nd:YAP) lasers; carbon dioxide (CO_2_) lasers; and diode lasers. The mechanism of action and application in dentistry of these lasers are summarized in Table 1. In this section, we will introduce the laser systems, respectively, with a particular focus on their application in endodontic treatment.

### 2.1. Er:YAG Laser

Erbium lasers were introduced into dentistry for the preparation of dental hard tissue, since their radiation is easily absorbed in water, and the instant evaporation of water inside the tissue causes a micro-explosion that can blast off tiny particles of hard substance [9]. As they have their own cooling system, there is little heat that is generated in the surrounding tissues [6]. The Er:YAG (2940 nm) laser, as a type of erbium laser, possesses a higher water absorbency in comparison with the CO_2_ and Nd:YAG lasers [10]. Due to the fact that its wavelength matches exactly the absorption peak of water, and it can be absorbed by hydroxylapatite, the Er:YAG laser is effective in ablation and hard tissue removal during root canal treatment, pulpotomy, etc. [11,12,13]. It should be noted that in clinical application, the pulse energy and repetition rate of the Er:YAG laser need to be controlled to avoid leaflets, cracks, and other side effects [11]. 

### 2.2. Er,Cr:YSGG Laser

The wavelength of Er,Cr:YSGG lasers is 2780 nm, which is very close to the absorption peak of water. Its applications and limitations in the context of endodontic treatment are similar to Er:YAG lasers, since both of them belong to the category of erbium-type lasers. Moreover, there are studies that have demonstrated that when a Er,Cr:YSGG laser irradiates the dental tissue with water spray, the temperature will barely increase due to the complete elimination of the friction source that would otherwise generate heat. At the same time, the cutting efficiency will also be improved [14]. 

### 2.3. Nd:YAG Laser

The Nd:YAG laser is a free-running pulsed-wave laser that emits light at 1064 nm. Different from the Er:YAG laser, the absorption of the Nd:YAG laser in water is low, with energy scattering and penetration of the adjacent biological tissues [15]. It can be applied to root canal irrigation [16,23]. However, its energy and its ability to cut hard tissue are not as good as the Er: YAG laser due to its wavelength. Thus, during clinical practice, the output power should be accurately mastered, and the irradiation time should be controlled, to protect the surrounding tissue.

### 2.4. Nd:YAP Laser

The Nd:YAP laser is emitted in the near-infrared at 1340 nm. It is easily absorbed by dark materials and metals, and its absorption by water is 20 times more than the Nd:YAG laser. The flexible fiber optic of the Nd:YAP laser can transfer energy in curved root canal surgery [17]. As such, several studies on this topic have demonstrated that the Nd:YAP laser can be utilized to eliminate the smear layer that is attached to root canal walls [18,24]. Furthermore, it should be used in pulsed mode with resting periods to avoid thermal damage to the surrounding tissues [25]. 

### 2.5. CO_2_ Laser

The first 10,600 nm CO_2_ gas laser was created in 1964. In addition, the CO_2_ laser at this particular wavelength is widely used in medicine and dentistry. Unlike the Nd:YAG laser, it is also easily absorbed by enamel and dentin. However, CO_2_ laser irradiation can lead to cracks on the enamel surface, which may encourage caries development along the crack lines [19]. Due to the fact that it can control haemorrhage, the CO_2_ laser is utilized in direct pulp capping [20]. 

### 2.6. Diode Laser

The diode laser is a type of low-power output laser. When used as a method for treatment, it is usually applied at a wavelength of 810–980 nm [4]. The diode laser can achieve a higher water absorption inside the dental hard tissues than the Nd:YAG laser, which means it can work on the microorganisms inside the dentinal tubules due to its greater penetration [21]. On the other hand, the diode laser exhibits considerable divergence, which results in poor optical performance. As a result, it can be effective in root canal treatment to eliminate the microorganisms from root canals, and reduce post-operative endodontic pain [22]. 

## 3. Lasers in Root Canal Treatment

The main pathogenic factor regarding endodontic diseases and periapical diseases is microorganism infection in the root canal systems. Root canal treatment is the most effective and widely used method for controlling infection, promoting periapical healing, and avoiding reinfection, through the steps of root canal shaping, disinfecting, and filling. 

### 3.1. Root Canal Shaping

Currently, root canal shaping is typically performed with hand and rotary instruments. The smear layer produced during the procedure is expected to be removed from the root canal walls. In addition, the bacteria that existed in it will undoubtedly affect the final effectiveness of the therapy [26]. Following the development of lasers, it was found that laser irradiation could help shape root canal walls, and remove the smear layer. The mechanism of lasers in the context of root canal shaping is that laser irradiation can vaporize water in dental hard tissues and ablate the surrounding tissue, so that the dentinal tubules will be opened, and the smear layer will thus be removed. 

In respect of this, an in vitro study demonstrated that the Er:YAG laser could ablate dental hard tissues, open dentinal tubules, and clear the smear layer when the distance between the dentin and the tip was close. Not only that, the Er:YAG laser can also reduce the risk of dentine suture formation, thus avoiding root fractures [12]. Similarly, Samiei et al. [27] conducted a comparative study of root canal preparation with and without the Nd:YAG laser in vitro, thereby selecting sixty single-rooted human premolars and dividing them into four groups according to different preparation methods. In group 1, conventional K-files and step-back techniques were used for tooth preparation. In group 2 and 3, the teeth were prepared using the Nd:YAG laser and rotary NiTi instruments, respectively. In group 4, the Nd:YAG laser and NiTi instruments were combined to prepare the teeth. The results showed that among the four groups, group 4 resulted in the best cleaning efficiency of the canal walls. Indeed, it was even better than the traditional K-files and step-back technique.

### 3.2. Root Canal Irrigation

All this time, the cleaning and antisepsis of root canals—which is of key importance to a successful root canal treatment—primarily depends on two procedures, namely mechanical instrumentation, and the disinfection of irrigates. However, when considering the fact that the anatomy of the root canal system is extraordinarily complicated, canal shaping performed with instrumentation is largely regarded as a means of creating access to the apical anatomy in the present [28]. Thus, in this regard, irrigation does play a key role in the context of infection control [29].

Traditional root canal therapy uses syringe and needle irrigation (SNI) in order to remove debris and biofilms, which is still the most favored form of irrigation protocol in clinical practices [29]. Despite its convenient and inexpensive nature, SNI also possesses certain inevitable shortcomings, such as being difficult to standardize and control [30], as well as disabling greater penetration into irregular anatomical structures [31]. Due to the limitations of conventional SNI, innovative techniques in order to help improve irrigation efficiency have been developed, including ultrasonic activation, sonic activation, and laser activation [32].

Laser-activated irrigation (LAI) was applied as an adjunctive method for root canal irrigation many years ago [33]. The mechanism of LAI uses fiber tips to generate small cavitation bubbles in irrigation solutions, whose volumetric oscillation can result in high-speed fluid motion, the making of biofilms, as well as other contents in the root canals, moving vertically. Due to these continuous rapid movements, the contents that are attached to root canal walls fall off, and are finally flushed out of the canals [34].

Introduced for the purposes of clinical use in 2012, photon-induced photoacoustic streaming (PIPS) is an example of one of the emerging LAI techniques. When compared with conventional LAI methods, it renders features of low energy (10 or 20 mJ) and short pulse length (50 μs) [34]. Furthermore, this method also possesses the ability to prevent thermal damage to the root canal walls and the periapical tissue, by placing its working tip in the crown side of root canals [35]. Shock wave-enhanced emission photoacoustic streaming (SWEEPS) was created in order to increase the debriding efficiency of the PIPS method [36]. The working mechanism of SWEEPS is similar to extracorporeal shock wave lithotripsy. As the cavitation bubble begins to collapse, a second pulse is transmitted through the liquid, thereby causing a second cavitation bubble. Then, the second cavitation bubble accelerates the collapse of the first one, thus forming a violent collapse and finally emitting a shock wave. In addition, the collapsing secondary cavitation bubbles that are close to the root canal walls also emit shock waves, thereby removing the debris attached to the walls [37].

Accumulated hard tissue debris is a type of byproduct from root canal shaping, which should be removed during irrigation. A comparative experiment used 30 extracted human mandibular molars to compare the effectiveness of three different auxiliary means for removing accumulated hard tissue debris, including ultrasonically activated irrigation, PIPS, and SWEEPS. In addition, the evaluation was performed by microcomputed tomography. The PIPS and SWEEPS groups were performed with special fiber tips (PIPS 600/9 and SWEEPS 600) using a 2940 nm Er:YAG laser. The results showed that there was less remaining debris after SWEEPS than after ultrasonically activated irrigation and PIPS, especially in regard to complicated structures, such as isthmuses [36].

As for the elimination of biofilm and calcium hydroxide, multiple studies have shown that LAI performed significantly better than SNI [31,38,39]. The ability of the Er:YAG laser for the purposes of biofilm removal was explored in a study using pig models. In addition, the remaining bacteria level after irrigation in the LAI group was clearly lower than that of the SNI group [40]. 

Nonetheless, the comparison between the efficiency of ultrasonically activated irrigation and LAI, engendered different results and views. The results of a comparative study, which used transparent resin blocks in two standardized root canals as the test models, demonstrated that LAI using a 2940 nm Er:YAG laser resulted in greater biofilm-mimicking hydrogel removal than ultrasonically activated irrigation [41]. Another in vitro study performed by Liu et al. [42] with thirty-eight mature single root canal premolars showed a different result. They found that the efficiency of passive ultrasonic irrigation for the purposes of bacteria removal presented more advantages in the coronal and middle thirds of the root canals when compared with LAI using a Nd:YAP laser, while both of them resulted in similar effects in the apical third. Similar to accumulated hard tissue debris removal, none of these adjuvant procedures could completely clear away biofilm components in root canal systems [43]. There are also some limitations to LAI, such as increased apical extrusion. The apical extrusion of debris, pulp tissue, solutions, bacteria, and their byproducts, is one of the causes of post-operative inflammation and pain, which can delay the healing of periapical tissue [44]. Certain investigations have proved that irrigation activated by the Er:YAG laser or the Nd:YAP laser caused more extrusion of debris when compared to needle irrigation [44,45].

### 3.3. Fiber Posts Surface Treatment

When the coronal loss is so severe that the remaining dental hard tissue cannot offer adequate support, while the teeth should be preserved for functional or aesthetic reasons, dentists will use fiber posts during endodontic treatment as intracanal retainers in order to disperse the forces transmitted to the roots, as well as to provide better retention and support [46]. Due to its superior high bonding strength and flexural strength, glass fiber posts have been applied for this purpose for over 20 years, with a 95% annual survival rate [47].

The lifespan of endodontically treated teeth using fiber posts is connected with the effectiveness of bonding among the posts, the dentin, and the resin cement, due to the fact that debonding is a major cause of failure [48]. In order to improve the therapeutic effect and to better prolong the longevity of treated teeth, researchers have studied many methods in order to enhance the bonding strength. Laser irradiation in order to better roughen fiber post surfaces is one such strategy. 

Gomes et al. [48] conducted an in vitro study with thirty-two mandibular bovine incisors and drew a conclusion that whether laser irradiation could improve the push-out bond strength (PBS) appeared to depend on the laser system. The pretreatment of glass fiber posts with an Er:YAG laser or a 980 nm diode laser did not increase the PBS in comparison with a silane control group, while the group pretreated with the Er,Cr:YSGG laser significantly favored the PBS. 

As for quartz fiber posts, an in vitro experiment showed that the irradiation of the Er,Cr:YSGG laser increased the PBS between quartz and dentin; additionally, an output power of 1.0 W was recommended in order to minimize the damage, and to maximize the PBS [49]. Furthermore, it was proved in another in vitro study that the irradiation of the Er:YAG laser helped increase the PBS between quartz fiber posts and the resin cement, while the Nd:YAG laser irradiation did not work as intended [50]. 

The controversial efficiency of fiber post surface treatment with lasers varies with different laser systems, and fiber post materials. Further research should be conducted in order to determine whether the laser’s energy, frequency, potency, pulse duration, irradiation time, and acting distance, influence the results of fiber post surface treatment. 

### 3.4. Filling Materials Removal

Completely removing the filling materials in root canals is an essential step in retreatment. This is due to the fact that the remnants can breed bacteria and block the apical area of root canals, thereby leading to retreatment failure. However, the filling materials are placed deep and dense in order to exert the biological effects fully around the periapical area, which renders the complete removal of the remnants a great challenge [51]. SNI, passive ultrasonic irrigation, and lasers are commonly used to remove the filling materials. It was reported that compared to SNI and passive ultrasonic irrigation, the activation of 2.5% NaOCl and 17% EDTA with an Er:YAG laser significantly increased the removal efficiency of the residual iRoot SP and gutta-percha after NiTi mechanical operation [52]. In addition to the Er:YAG laser, the Nd:YAG laser was also shown to be useful for the purposes of filling material removal [53,54].

We summarized the research on the application of lasers in root canal treatment. Meanwhile, details and parameters of the samples in the in vitro experiments and the application of the laser devices are listed in Table 2.

## 4. Lasers in Vital Pulp Therapy

The preservation of pulp vitality is a critical target during endodontic treatment. In contrast with root canal treatment, vital pulp therapies, including pulp capping and pulpotomy, are available as options, offering less invasion and a higher cure rate for the carious exposed pulps [55]. Pulp capping is a therapy that is used in order to place the healing agents directly or indirectly on the pathologically exposed pulp. Additionally, pulpotomy entails the removal of the coronal infected portion of the pulp, and the preservation of the Hygeian radicular pulp [56].

Mineral trioxide aggregate, calcium hydroxide, and formocresol, are all commonly used drugs. For the purposes of pulp capping treatment, calcium hydroxide has been widely used for decades. However, it must be noted that this practice often results in diverse and unpredictable outcomes [57]. Further, Mineral trioxide aggregate was proved efficacious, but is relatively expensive [56]. Meanwhile, the cytotoxicity, carcinogenicity, and mutagenicity of formocresol may affect the long-term results [58]. Therefore, optional choices, such as laser-assisted treatment, are also acceptable. 

The utilization of laser irradiation could accelerate the formation of the fibrous matrix and the dentin bridge. Further, the use of laser irradiation could also promote the expression of lectins and collagens in the exposed dental pulp tissues, thereby resulting in wound healing in respect of the pulp [59]. A low-power laser has been demonstrated to activate a growth factor complex for dentin regeneration in the pulp capping teeth of rat models [60]. While low-level lasers could modulate inflammation and the healing process, high-level lasers could promote pulp healing by the means of increasing the temperature [61]. In addition, low-level laser therapy as an adjuvant alternative to pulpotomy has also shown a satisfactory effect in the research [62].

### 4.1. Lasers in Pulp Capping

Clinical research on laser applications in pulp capping was carried out. A pilot study proved that the diode 808 nm laser-assisted procedure, including hemostasis and decontamination, could enhance the outcomes of pulp capping treatment for the purposes of carious exposures [63]. Similarly, Yazdanfar and colleagues combined a diode 808 nm laser and a resin-modified tricalcium silicate (TheraCal LC). Through performing this research, the laser TheraCal group was found to possess a thicker dentin deposit and less sensitivity to cold stimulation when compared with the TheraCal group [64]. Calcium hydroxide paste, resin-based tricalcium silicate material, an Er,Cr:YSGG laser, and a Gr Laser were used, singly or in combination, for treating the exposed pulp. The results from a 6-month follow-up period, indicated that laser irradiation when combined with pulp capping agents was recommended for the purposes of direct pulp capping treatment [65]. According to a systematic review, 80% of the studies showed adjunct laser therapy had an advantage over conventional therapy alone in maintaining pulp vitality. The outcomes appeared to be influenced by extra factors, including pulpal bleeding conditions, causing pulp exposure, and adjacent contamination [61]. 

In this respect, through various histological studies in animals, a consensus was reached. The histopathology of pulpal and periapical tissue after direct pulp capping with a low-level 980 nm laser application was analyzed. The laser-aided group showed mild inflammation, organized odontoblasts, thicker predentin in the pulp tissue, and more fibrosis in the periapical area. The histologic sections were evidence that lasers could enhance healing after direct pulp capping [66]. However, there has been some debate on the tissue response to lasers. Suzuki et al. reported that CO_2_ laser irradiation could inhibit the formation of dentin bridges by creating heat-denatured tissue around the exposed pulp. The thermal-induced denaturation was aggravated in proportion to the intensity of the lasers. Therefore, the selection of the wavelength or the frequency of the irradiation was deemed to be noteworthy [20]. In general, lasers could be regarded as a potential adjunct tool for maintaining pulp vitality; however, the suitable devices and wavelength remain to be studied.

### 4.2. Lasers in Pulpotomy

In conventional pulpotomy treatment, the dentist amputates the putrescent coronal pulp with a round bur at a low speed, and removes this portion with a spoon excavator. After flushing and drying the pulp chamber, moist cotton pellets with ferric sulfate, formocresol, or other coagulative agents, are placed in it [67]. After complete hemostasis, the utilization of lasers could participate in the procedures by ablating the pulp to the canal level, as well as in promoting the coagulation and healing of the pulp [58,67].

The clinical success of the pulpotomy treatment for primary teeth could be influenced by laser application. In order to face the challenges of various degrees of cooperation and the behavior management of children undergoing dental procedures, the laser was introduced on account of its low noise, as well as the reduced contact between tooth and mechanical instrumentation, which was especially attractive to pediatric dentists [68]. As such, the effects of an Er:YAG laser utilized in pulpotomy in primary molars with deep caries decay were evaluated. The 1.3 mm laser fiber tip was put at a 1 mm distance from the pulp tissue at the root canal orifice for irradiation. In contrast to conventional low-speed ball drilling pulpotomy, the Er:YAG laser group possessed a shorter hemostasis time and less total treatment time, as well as better clinical efficacy during long-term follow-ups [13]. Low-power or high-power diode laser irradiation was also considered as an effective alternative for primary teeth treatment. Further, concerning mineral trioxide aggregate and laser therapy, Mineral trioxide aggregate group and low-power diode laser mineral trioxide aggregate group reached a clinical success rate of 100% in a randomized trial on deciduous molars pulpotomy, while 87.5% of the high-power diode laser mineral trioxide aggregate group avoided clinical failure [69]. In another study, low-level laser therapy was compared with formocresol for the purposes of pulpotomy in primary teeth, and was found to possess advantages, albeit by a narrow margin [70]. As for permanent teeth, a clinical and in vivo trial were conducted that applied an Er,Cr:YSGG laser with a mineral trioxide aggregate in unison for the purposes of treating permanent immature molars. It was found that the success rate of this joint use was slightly higher than applying mineral trioxide aggregate alone [71]. 

Clinical evidence shows that lasers could perform as an alternative for primary teeth pulpotomy, with better patient compliance and outcomes. However, the selection of the devices and wavelength of the lasers still needs to be researched. Meanwhile, as certain trials showed no statistical significance, more survey samples and longer follow-up periods are expected and encouraged for the purposes of further research. The parameters and details of the above-mentioned clinical trials on the application of lasers in vital pulp therapy are listed in Table 3.

## 5. Lasers in Pain Management in Endodontic Treatment

Pain is a major concern of patients; the prevention and management of pain during or after endodontic treatment are often critical for the patient’s comfort and quality of life [22]. Endodontic diseases, including irreversible pulpitis, apical periodontitis, and acute apical abscess, are common reasons for dental pain [72,73]. Pre-operative pain and persistent pain are important factors related to endodontic treatment failure [74]. Meanwhile, as a common complication occurring after endodontic treatment, post-operative endodontic pain was reported to be at a prevalence of up to 40%, and the control of post-operative pain was regarded as a crucial target of endodontic therapies [75,76]. In addition, the etiology of pain is related to the chemical, mechanical, or microbial factors that could damage pulp or periapical tissue [77]. For instance, tooth debris, intra-canal dressings, and microorganisms could irritate peri-radicular tissues and lead to inflammation [78]. Additionally, the nociceptor is triggered through inflammatory mediators (leukotrienes, prostaglandins, bradykinin, etc.), thereby resulting in unexpected pain [79]. Further, microorganisms are recognized as the main reason for post-operative pain [77]. The conventional solution to break through the microbial challenge, including combining root canal disinfectants and mechanical apparatus, has become the representative procedure in clinical practice [80]. Unfortunately, certain drug-resistant bacteria are hard to eliminate, as they can settle in filled root canals for a long period of time [5]. Meanwhile, such microorganisms have been demonstrated to penetrate dentine via the dentinal tubules [81]. Therefore, traditional chemomechanical preparation could engender a constant risk of inflammation, and post-operative pain. This is all the while lasers could reach the bacteria in the deeper dentin layers without damaging the tooth structure [82].

Lasers were found to reduce pain-inducing substances (such as substance P, histamine, dopamine, and prostaglandins), as well as increase the synthesis of lymphokines, immunoglobulins, anti-inflammatory prostaglandins, and beta-endorphins [83,84]. The in vivo research showed certain laser-based therapies could lead to the inhibition of neural activity, conduction blockage, disruption of fast axonal flow, and the downregulation of signals from nociceptors, thereby showing significant potential to relieve pain [85]. Currently, with the advancements in laser science, novel laser-based therapies (i.e., conventional laser use, photobiomodulation therapy, and antimicrobial photodynamic therapy) have been applied for the purposes of addressing pain alleviation in endodontics [86].

Clinical trials have been carried out for different endodontic diseases. Patients with apical periodontitis suffer from typical spontaneous pain or pain on percussion when undergoing endodontic treatment. The associated pain intensity may be mitigated after the sources of inflammation and infection are removed using proper procedures. Lasers with different wavelengths or applying methods have also been introduced to treatment modalities for the purposes of decontamination and debridement of the root canal system. Yeon-Jee et al. utilized a 1440 nm Nd:YAG laser in order to treat teeth with symptomatic apical periodontitis via intracanal irradiation. The 300 μm fiber optic tip was dangled at the apical 3 mm level of the root canals for 10 s in the experimental group. By combining the assessment of the patient’s subjective pain level and the quantification of pain-related substances, they found that the irradiation group performed significantly better at the alleviation of pain and reduction in substance P, calcitonin gene-related peptide, and matrix metalloproteinase levels than what was found in the control group [87]. Another study utilized a 940 nm diode laser in the context of root canal disinfection, following root canal filling removal and preparation. The energy (1 W, 2 mm/s) was continuously delivered via a 200 μm diameter fiber tip at working length. In contrast to a placebo group, patients underwent slightly less post-operative pain and less analgesic intake in the laser disinfection group [22]. Meanwhile, a randomized control trial in relation to the 980 nm diode laser’s effect on necrotic teeth was carried out. In this trial the laser was considered as a successful adjunct to more traditional treatments for necrotic cases [88].

Photobiomodulation therapy is an alternative therapeutic strategy for dealing with safe and non-invasive features in order to reduce pain and inflammation [89]. Photobiomodulation therapy involves low-dose light treatments utilizing varieties of light sources, including lasers, broadband light, and LEDs [90]. In the clinical research of Lopes and colleagues, photobiomodulation therapy with 808 nm indium–gallium–aluminum laser irradiation was performed immediately after endodontic treatment on patients with irreversible pulpitis on the corresponding gingival point of the root apex of their lower molar teeth. They found that, following this approach, the prevalence of postoperative pain in the experimental group decreased significantly [91]. In another study that involved the inclusion of a placebo group, low-level laser therapy was also considered a benefit in respect to post-operative pain reduction for a patient with apical periodontitis [92].

Photodynamic therapy, also known as antimicrobial photodynamic therapy, performs using antimicrobial properties via an interaction between a light source at a specific wavelength and a photosensitizer [93]. Furthermore, through a clinical study focused on the influence of photodynamic therapy on pain after the treatment of teeth with necrotic pulps, there was a conclusion that aligned with this notion. Methylene blue was utilized as a photosensitizer and immersed in the canals before the operator inserted the optical fiber (100 mW, 3 min, 18 J) into the working length. The subjects were also required to document their pain perception via a visual analogue scale. The results show that photodynamic therapy notably relieves the post-operative pain of necrotic teeth [94].

However, clinical research depends on various factors, for instance, the effects of laser irradiation in relation to biological tissue, the complex causes of post-operative pain, and the individual differences in respect to pain perception. Recently, Fatma and colleagues paid attention to the lasers’ efficiency in respect of the post-operative discomfort of a patient with irreversible pulpitis and necrotic pulps. In their investigation, Nd:YAG and diode lasers were applied as adjuvant to the standard procedures, while a visual analogue scale was recorded in order to quantify the pain intensity. However, the use of the Nd:YAG and diode lasers showed no obvious improvement or deterioration of the pain level in patients with irreversible pulpitis and necrotic pulps. Furthermore, they advised that the body’s immune system will take major responsibility for healing as long as the contaminant removal and dense sealing have been executed properly [95]. 

## 6. Lasers in Dentinal Hypersensitivity Treatment

The definition of dentinal hypersensitivity is the pain arising from the exposure of open dentinal tubules to various stimuli including thermal, chemical, or tactile factors [96]. The stimulation could activate A-δ nerve fibers in the dentinal tubules via the hydrodynamic mechanism, thereby resulting in the onset of the type of annoying pain that is sharp, short, and well-localized [97]. Indeed, attrition, abrasion (especially the wedge-shaped defect type), erosion, abfraction, and gingival recession, could all be the etiologies of dentinal hypersensitivity [97]. Meanwhile, tooth decay could destroy the integrity of dental hard tissue and lead to dentinal hypersensitivity; the altering of structure accompanied by thermal, chemical and mechanical stimulus, could result in pulpal inflammation [98]. As such, conventional therapy, such as self-applied and clinical treatment, rely on materials and drugs that could occlude dentinal tubules or block nerve activity [99]. In this respect, topical desensitizing fluorine pastes and sealants are common choices [96]. Some patients turn to endodontic treatment due to the dental pain related to dentinal hypersensitivity. Irreversible interventions, such as root canal treatment, could be taken into consideration after trying all conventional alternatives [98]. Recently, a study has shown that lasers could promote dentinal tubule occlusion physically through the alteration of the surface, or reduce sensitivity via protein coagulation [97]. In addition, laser irradiation has also been introduced as a non-drug and non-invasive therapy, and could be combined with classic desensitizing interventions.

Numerous clinical research studies have compared laser-based therapy with drug-based protocols. Sgreccia et al. performed a randomized clinical trial, aimed at evaluating the low-power laser and potassium oxalate gel treatment for the purposes of cervical dentin hypersensitivity. Although the potassium oxalate resulted in immediate symptom reduction, the GaAlAS laser (100 mW, 808 nn, 60 J/cm^2^) irradiation reached similar consequences at the end of the respective therapies [100]. In addition, a study compared the therapeutic efficacy of an Nd:YAG laser with settings at 60 mJ, 2 Hz and 0.64 W, and a new varnish with casein phosphopeptides. The 300 μm fiber was put 6 mm away from the exposed dentin. A clear remission of dentin hypersensitivity and insignificant differences between the two therapies was also indicated [101]. Furthermore, oral health-related quality of life was measured by Lima and colleagues. A total of 80% of the participants, after the application of a 795 nm laser diode with the power of 120 mW or cyanoacrylate treatment, reported an improvement in their conditions via their oral health impact profile (OHIP)-14 subscales [102]. Moreover, different types of lasers possess distinct performances in respect to dentinal hypersensitivity treatment. Another study rendered a comparison of Er,Cr:YSGG (2780 nm wavelength, 140–200 μs pulse width) and diode lasers (940 nm wavelength, 1 min irradiation times) through a split-mouth randomized clinical trial. In respect of this, it was reported that the preponderance of an Er,Cr:YSGG laser within a one-month post-operative time interval was possible [103].

In parallel, several researchers have accounted for the optimal parameters of laser treatment via in vitro studies; further, this was achieved by compromising between therapeutic effect and pulp safety. Zhuang and colleagues analyzed the performance of an Er:YAG laser with different parameters in the context of dentin tubule occlusion via scanning electron microscopy. Meanwhile, the assessment of the intrapulpal temperature stabilization and pulp tissue morphological invariability was also conducted. In addition, the extracted human molars after Er:YAG laser irradiation at 0.5 W (167 J/cm^2^) showed superior dentin tubule occlusion, less intrapulpal temperature change, and hardly any morphological alterations of the pulps [104]. The dentine of different thicknesses was irradiated with a Nd:YAP laser in an ex vivo study; whilst conducting this, they found that a Nd:YAP laser at a PD of 356 W/cm^2^ had a significant effect on the diameter change in dentinal tubule orifices and caused less of an increase in temperature [25].

However, a recent meta-analysis by Mahdian et al. involved the selection of randomized controlled trials with subjects over 12 years old. They confirmed the safety of laser therapy, but presented limited evidence in respect of its function for pain intensity improvement [105]. Further, double-blinded trials considering the placebo effect are still required and are vital for the purposes of further investigation.

## 7. Future Directions

As laser technology advances, its application in dentistry is becoming more widespread. However, there are still many potential applications that remain unexplored. 

### 7.1. Broken Files Removal

There are two ways to remove separated instruments that are stuck in the apical region, either by directly irradiating the instrument itself with lasers to melt it; or by irradiating the dental tissue around the instrument with lasers in order to create a bypass, which is then removed by other instruments. However, both methods have non-negligible drawbacks, such as: melting metal instruments requires high-energy lasers, which can cause thermal damage to the adjacent tissues; melting the tissues around the instruments does not require such high-energy, but when used for curved or thin-walled root canals, it can easily cause lateral perforation. Despite these limitations, Yu et al. [54] conducted an in vitro study to investigate the ability to remove broken files from root canals with pulsed Nd:YAG laser, and adopted the method of creating a bypass in this in vitro study. They reported that an Nd:YAG laser could be useful for the purposes of broken file removal if measures were taken to control the temperature increase, such as combined pressured air and water spray. In recent years, few studies have been conducted with respect to addressing the removal of broken files with lasers. However, it must be stated that this method is potentially clinically applicable and deserves more in-depth study. 

### 7.2. Fiber Posts Removal

When considering the aesthetics and survival rate of the current root canal treatment, various measures are taken in order to enhance the bonding strength between the fiber, resin cement, and dentin. The semi-transparency of glass fiber posts is an added advantage, compensating for the aesthetic shortcomings of carbon fiber posts, thereby rendering endodontically treated teeth more similar to the appearance of natural teeth [106]. However, when it comes to retreatment, it can be a great challenge for dentists to remove the translucent glass fiber posts that are similar in appearance to the dentin and closely adhere to it, whilst preserving as much of the dental tissue as possible [47]. Moreover, more and more patients possess a need for retreatment after failing root canal treatment, due to the fact that there is a certain failure rate in respect of root canal therapy, and patients increasingly desire to keep their own teeth [107].

Clinically, glass fiber posts are often removed by mechanical or ultrasonic methods. However, these mechanical methods can cause obvious loss of tooth structure; additionally, the heat generated during the process can also cause damage to the dental tissue. Compared to mechanical methods, ultrasonic methods can reduce tooth structure loss, but they can cause heat and microcracks, thus increasing the risk of tooth fracture [47]. Recently, lasers have been used for the removal of fiber posts. Deeb et al. [47] used the Er:YAG laser (2 W, 15 Hz, 135 mJ, 50 µs) with an endodontic tip in SWEEPS mode in order to remove fiber posts in vitro, and found that the Er:YAG laser could effectively remove them. Meanwhile, they found that the temperature rises and microcracks caused by lasers were less than those caused by ultrasonic instruments. Further, the removal speed achieved by utilizing the lasers was five times faster than the ultrasonic device. In another in vitro study, it was shown that the Er,Cr:YSGG laser (2.5 W, 20 Hz, MZ5 endodontic tip) can effectively remove the glass fiber posts and at the same time retain more dental tissue, according to a micro-CT evaluation [108]. However, there are few studies that are focused on fiber post removal by lasers; as such, the specific effects of this technique have yet to be verified. 

### 7.3. Root Development Acceleration

Trauma and decay can cause necrosis of the pulp tissue, which can lead to the obstruction of normal root development and an open apex [109]. MTA is most commonly used in order to induce an apical barrier, which is considered to be the gold standard for the treatment of teeth with an open apex and pulp necrosis [110]. Research has demonstrated that the combination of the GaAlAs diode (810 nm) laser and MTA can accelerate dentinogenesis and apexogenesis of teeth in rats and dogs, thus speeding up treatment [109,110,111,112]. In the study by Bahman et al. [111] three healthy 4–6-month-old dogs with similar living conditions were selected, and 36 teeth involved in this in vivo study were divided into two groups, calcium hydroxide with GaAlAs diode laser (470 mW/cm^2^, 59 J/cm^2^) irradiation and calcium hydroxide without laser irradiation, respectively. According to the findings of this study, the combination of calcium hydroxide and laser irradiation had beneficial effects on the apexogenesis process. Therefore, lasers possess the potential to be utilized as an adjunctive method to promote the root development process. However, more clinical studies and several animal studies are needed in order to validate their effectiveness in the future.

## 8. Conclusions

Lasers stimulate atoms or molecules in order to emit a specific wavelength of light and amplify it, resulting in coherent, monochromatic, well controlled, and precisely directed light beams. These characteristics render them useful in a wide range of fields, such as transmission, imaging, measurement, etc. When a laser interacts with biological tissues, it produces special biological effects, including thermal, photochemical, electromagnetic, and biological stimulation effects, which are the basis of its use in medicine. The rapid development of laser technology can usher in a new era for the diagnosis, prevention, and treatment of multiple diseases. More and more attention has been directed to its application and prospects in dentistry, and its clinical implications are mainly divided into two parts: hard tissue applications and soft tissue applications. Caries prevention, cavity preparation and dentinal hypersensitivity treatment are examples of hard tissue applications of lasers in dentistry, while soft tissue applications include gingivectomy, gingivoplasty, labial frenectomy, lingual frenum release, mucositis, and so on. In recent years, the literature has fully proved that laser treatment is an effective adjunctive method for endodontic treatment.

In this review, we summarized the novel applications of lasers in endodontics and certain common dental procedures, regarding laser-based therapy as a potential option for dental treatment. However, the review is narrative; thus, no comprehensive analysis of the literature has been performed. Some conclusions related to the ideal type of laser and parameters for certain clinical purposes still required deliberations. Moreover, there are certain drawbacks to lasers, including the potential for causing erythema, skin hyperpigmentation, thermal injury, and eye injury. As a result, the clinical use of lasers must be performed by professionally trained dentists, which is also a barrier to their application in clinical practice. Another major drawback that hinders the application of lasers is the cost factor.

There is no doubt that, in the future, lasers will be used more frequently in endodontics. As a result, future research in this field will focus on the issue of how to overcome the shortcomings, prevent treatment-related accidents, and apply it to more clinical examinations and treatments.

## Figures and Tables

**Table 1:** Lasers in endodontics.

Laser Device	Wavelength	Characteristics	Application	Reference
Er:YAG laser	2940 nm	(1) Easily absorbed into hydroxyapatite crystals (2) Water evaporation results in small degrees of heating and micro-explosions (3) Ablation and hard tissue removal	(1) Root canal treatment (2) Pulpotomy	[9,10,11,12,13]
Er,Cr:YSGG laser	2780 nm	(1) Similar to Er:YAG lasers (2) Almost no heat and also high cutting efficiency with water sprays	Similar to Er:YAG laser	[14]
Nd:YAG laser	1064 nm	(1) Energy scattering and penetration in adjacent biological tissues	(1) Root canal irrigation	[15,16]
Nd:YAP laser	1340 nm	(1) Easily absorbed into dark materials, metals, and water (2) Transfers energy into curved root canals	(1) Eliminates the smear layer on root canal walls	[17,18]
CO_2_ laser	10,600 nm	(1) Easily absorbed by enamel and dentin (2) Hemostasis	(1) Widely used in medicine and dentistry such as direct pulp capping	[19,20]
Diode laser	810–980 nm	(1) Great penetration (2) Works on the microorganisms inside dentinal tubules	(1) Eliminates the microorganisms in root canals (2) Reduces post-operative endodontic pain	[4,21,22]

**Table 2:** In vitro experiments on the application of lasers in root canal treatment.

Laser Device	Application Parameters	Characteristics of Samples	Operations of Laser Applications	Reference
Er:YAG laser	30 m J, 20 pps, water flow of 5 mL/min	Six single-rooted human teeth	Three times irradiation for 10 s	Kokuzawa et al. 2012 [12]
Nd:YAG laser	Output power of 35 W, a wavelength of 1.06 µm	Sixty single-rooted human premolars	Fiber optic device was placed in root canals to reach the working length	Samiei et al. 2014 [27]
Er:YAG laser	0.6 W, 15 Hz, 40 mJ	3D-printed isthmus models with artificial biofilms	The fiber tip was positioned 2 mm above each root canal entrance, and activation was performed 2 × 30 s	Robberecht et al. 2023 [31]
Er:YAG laser	0.3 W, 15 Hz, 20 mJ per pulse	Thirty mandibular first and second molars	Fiber tips were put into the access cavity for 3 × 30 s	Yang et al. 2020 [36]
Diode laser (970 mm)	The maximum output power of 14 W	Sixty-three monoarticular teeth	Four cycles of 10 s each of irrigation solution activated by a laser, with a 5 s break between each cycle	Cîmpean et al. 2022 [38]
Er:YAG laser	2.06 J/cm^2^, 15 Hz, 20 mJ	Eighty-four S-shaped endo training blocks	The irrigant was activated for 30 s	Shi et al. 2022 [39]
Er:YAG laser	20 Hz, 50 μs, 20 mJ	Transparent resin blocks containing two standardized root canals	Tips put at the canal entrance for 3 × 20 s	Swimberghe et al. 2019 [41]
Nd:YAP laser	280 mJ, 5 Hz, and 1.4 W	Thirty-eight mature single root canal premolars	Tips were placed 2 mm from the working length and activated at 0–4 s, 13–17 s, and 26–30 s	Liu et al. 2022 [42]
Er:YAG laser	0.3 W, 15 Hz, and 20 mJ	Sixty extracted maxillary first molar teeth	Tips were placed into the pulp chamber and activated for 20 s	Arslan et al. 2018 [44]
Nd:YAG laser	1 W, 20 Hz and 50 mJ	Extracted human mandibular premolar teeth	Fiber tip was placed into the apical third of the root canal and activated for 10 s	Yıldız et al. 2020 [45]
Er:YAG laser; Er,Cr:YSGG laser; diode laser (980 mm)	Er:YAG laser (1.5 W, 10 Hz, 150 mJ, 100 μs) ; Er,Cr:YSGG laser (1.5 W, 10 Hz, 150 mJ, 140 μs); diode laser (1.5 W, continuous)	Thirty-two mandibular bovine incisors	Er:YAG laser (60 s, non-contact); Er,Cr:YSGG laser (60 s, non-contact); diode laser (15 s, contact mode)	Gomes et al. 2018 [48]
Er,Cr:YSGG laser	20 Hz frequency and 150 μs pulse duration	One-hundred-and-five fiber posts	Lasers were used in circumferential or longitudinal motion directions	Rezaei-Soufi et al. 2019 [49]
Er:YAG laser; Nd:YAG laser	Er:YAG laser (1.5 W, 10 Hz, 150 mJ); Nd:YAG laser (1 W, 10 Hz, 100 mJ)	Sixty-six quartz fiber posts	Nd:YAG laser (300 mm diameter laser optical fiber for 20 s); Er:YAG laser (pulse duration of 700 ms for 20 s)	Akin et al. 2014 [50]
Er:YAG laser	0.3 W, 15 Hz, 20 mJ, 50-µs pulse	Thirty-six extracted single-rooted teeth	Tip was placed in the access cavity for 40 s	Yang et al. 2021 [52]
Nd:YAG laser	900 mJ/pulse, pulse width 0.3 ms EC pulse, pulse frequency 5 pps	Forty single-rooted human teeth	Keeping the fiber tip approximately 0.5 mm away from the surface of the root canal obturation material	Anjo et al. 2004 [53]
Nd:YAG laser	1.0 W, 15 pps; 2.0 W, 20 pps; 3.0 W, 20 pps	Thirty-six extracted human single root canal incisors	3 s irradiation time, 1 s interval; 2 s irradiation time, 2 s interval; 2 s irradiation time, 2 s interval	Yu et al. 2000 [54]

Abbreviations: SWEEPS: shock wave-enhanced emission photoacoustic streaming; LAI: laser-activated irrigation.

**Table 3:** Clinical trials on the application of lasers in vital pulp therapy.

Vital Pulp Therapy	Laser Device	Application Parameters	Characteristics of Samples	Operation of Laser Applications	Reference
Pulp capping	Diode laser	1.5 W, continuous wave (hemostasis); 1 W, continuous wave (decontamination)	Ten patients underwent conservative treatment for deep caries in permanent teeth	2 s per 1 mm, vertical and horizontal movement (hemostasis); 2 mm per s, circular movement (decontamination)	Yazdanfar et al. 2014 [63]
	Diode laser	1.5 W, 90° tip angle, 2 s per area	Twenty anterior and posterior teeth of fourteen patients	Vertical and horizontal scanning movement on the exposure site	Yazdanfar et al. 2020 [64]
	Er,Cr:YSGG laser	0.5 W, 20 Hz, 140 ms pulse duration	Sixty teeth of sixty patients	Non-contact mode for 10 s	Yang et al. 2020 [65]
	CO_2_ Laser	0.003 J/pulse, 0.5 W	Twenty-eight third molar teeth of seventeen volunteers	Repeat mode for 15 s	Suzuki et al. 2019 [20]
Pulpotomy	Er:YAG laser	20 mJ/pulse, 0.3 W, 15 Hz	Three to six-year-old children with asymptomatic deep caries	1.3 mm laser fiber tip was put at a 1 mm distance from the pulp tissue at the root canal orifice	Wang et al. 2022 [13]
	Diode laser	Low-level laser therapy (660 nm, 200 mW); high-power diode laser (810 nm, 1 W)	Sixty-three primary mandibular second molars in children aged four to seven years old	Low-level laser therapy (laser probe in contact with occlusal surface and at a distance of 4 mm from the exposure site); high-power diode laser (fiber in contact with the pulp tissue)	Ebrahimi et al. 2022 [69]
	Er,Cr:YSGG laser	0.5 W, 20 Hz	Ninety caries-exposed permanent immature molar teeth in patients aged between six and fifteen years old	Tips on the hard tissue in non-contact mode for 10 s	Tozar et al. 2020 [71]
